# Biomaterial Hypersensitivity: Is It Real? Supportive Evidence and Approach Considerations for Metal Allergic Patients following Total Knee Arthroplasty

**DOI:** 10.1155/2015/137287

**Published:** 2015-03-25

**Authors:** Andrew J. Mitchelson, Craig J. Wilson, William M. Mihalko, Thomas M. Grupp, Blaine T. Manning, Douglas A. Dennis, Stuart B. Goodman, Tony H. Tzeng, Sonia Vasdev, Khaled J. Saleh

**Affiliations:** ^1^Division of Orthopaedics and Rehabilitation, Department of Surgery, Southern Illinois University School of Medicine, P.O. Box 19679, Springfield, IL 62794-9679, USA; ^2^Department of Orthopaedic Surgery & Biomedical Engineering, University of Tennessee, Memphis, TN 38017, USA; ^3^Clinic for Orthopaedic Surgery, Campus Grosshadern, Ludwig Maximilians University, 80539 Munich, Germany; ^4^Aesculap AG, Research & Development, 78532 Tuttlingen, Germany; ^5^Colorado Joint Replacement, Denver, CO 80210, USA; ^6^Department of Orthopaedic Surgery, Stanford University School of Medicine, Redwood City, CA 94063, USA

## Abstract

The prospect of biomaterial hypersensitivity developing in response to joint implant materials was first presented more than 30 years ago. Many studies have established probable causation between first-generation metal-on-metal hip implants and hypersensitivity reactions. In a limited patient population, implant failure may ultimately be related to metal hypersensitivity. The examination of hypersensitivity reactions in current-generation metal-on-metal knee implants is comparatively limited. The purpose of this study is to summarize all available literature regarding biomaterial hypersensitivity after total knee arthroplasty, elucidate overall trends about this topic in the current literature, and provide a foundation for clinical approach considerations when biomaterial hypersensitivity is suspected.

## 1. Introduction

Support for the theory of joint implant loosening caused by hypersensitivity reactions to metallic implant components was first presented in the mid-1970s [1, 2]. Studies were undertaken in response to clinical evidence of hypersensitivity reactions in patients after hip arthroplasty with metal-on-metal implants. Over the decades, this topic was examined in many publications. In a 2012 review of all available literature, Cousen and Gawkrodger established that first-generation metal-on-metal implants could cause sensitization of patients to the implant metals [3]. They also reported an association between metal sensitization and implant failure but did not establish a causal relationship [3]. A new generation of metal-on-metal implants is now available to surgeons. The widespread use of these new metal-on-metal implants, particularly in knee arthroplasty where their use is novel, raises questions regarding risks and benefits compared with more established implant options.

The theory that metal sensitivity and cutaneous allergic dermatitis develop in patients following implantation of metallic orthopaedic devices is supported by clinical and temporal evidence [4]. Reports also suggest that hypersensitivity to implant metals occurs in a considerable number of patients, although the prevalence of this phenomenon is not known [4, 5]. In a prospective study, the incidence of sensitization to metals in orthopaedic implants, as determined by patch testing, increased by 6.5% following hip and knee arthroplasty [6]. Similarly, in a review of hip arthroplasty, the rate of sensitivity to nickel, cobalt, or chromium was 25% in patients with well-functioning implants; this is more than twice the rate found in the general population [7]. In patients with a failed or failing hip prosthesis, the rate of metal sensitivity rises dramatically to 60%, six times that of the general population [7]. One study examined rates of metal sensitization in patients who had undergone total knee arthroplasty (TKA) and found a sensitization rate of 20% in the control group with no implant, 48.1% in the group with the stable implant, and 59.6% in the group with an unstable implant group [8]. Mihalko et al. performed an analysis of available prospective and retrospective studies regarding hypersensitivity reactions after total joint arthroplasty; the findings of this analysis are provided in Table 1 [6, 9, 10] and Table 2 [1, 2, 11–18]. The available evidence indicates a correlation between metallic orthopaedic implants, the development of metal hypersensitivity, and implant loosening.

## 2. Results

### 2.1. Implant Composition and Wear

Orthopaedic implants can be made of a variety of metallic, plastic, and/or ceramic elements. The metal components of knee prostheses are most commonly stainless steel, followed by cobalt-chromium-molybdenum (CoCrMo) alloys, nickel, titanium, Vitallium (Austenal Company), beryllium, vanadium, and tantalum [4, 25].

In a knee with an implant, metallic surfaces are freely exposed to synovial fluid. Contact with biologic fluid results in metal corrosion [3, 6, 26–31]. Cadosch et al. reported evidence of growth and differentiation of human osteoclast precursor cells occurring directly on surgical stainless steel, titanium, and aluminum [30, 31]. The mature osteoclasts then directly corroded the metal surfaces and released ions into the joint space [30, 31]. Free metal-ion compounds may then bind to endogenous proteins and form metal-protein complexes [4, 25]. These metal-protein complexes may subsequently initiate an immune reaction. Caicedo et al. provided evidence that the macrophage inflammasome pathway is activated by implant debris [28]. CoCrMo alloy debris has been shown to induce macrophage activation, stimulate secretion of interleukin-1 beta (IL-1*β*), tumor necrosis factor *α*, IL-6, and IL-8, and upregulate nuclear factor-*κβ* (NF-*κβ*) and downstream inflammatory cytokines [25]. Direct corrosion of metallic surfaces by osteoclasts similarly results in ion-induced secretion of proinflammatory cytokines [30, 31]. Although these inflammatory reactions are generally acute, implant debris in the periprosthetic region may in some instances result in a chronic inflammatory response [6].

### 2.2. Hypersensitivity Pathology

Exposure to metal ions can occur in a number of ways. Routine metal exposure in humans occurs through skin contact with jewelry, cell phones, clothing fasteners, and leather and through occupational exposure, dental filings, and medical implants [32]. Individuals are further exposed to trace metals through smoking and in cosmetics, food, and drinking water [33–35]. Nickel is the metal that most often leads to a hypersensitivity reaction; studies place the prevalence of nickel sensitivity in the general population between 8 and 25% [36–39].

Sensitization to metal is known to occur independently of the mechanism of exposure [4]. As previously mentioned, metal-ion exposure produces an adaptive immune response wherein macrophage activation leads to development of a delayed-type hypersensitivity reaction [4, 5, 7, 26, 40, 41]. In arthroplasty patients with a metal allergy, studies have shown elevated levels of interferon gamma and IL-6 [40, 41]. Similarly, in patients with nickel allergy, complicated joint implants were associated with substantially elevated levels of IL-17 when compared to uncomplicated implants [42]. These studies provide further evidence of delayed-type hypersensitivity reactions in response to implanted metal. Additionally, periprosthetic immune responses to metal have been shown to display characteristics of type I hypersensitivity reactions [40]. There is increasing support that metal hypersensitivity can be caused by orthopaedic arthroplasty components. Thyssen et al. presented a list of objective criteria which, when present in a patient, support a causative association between implant-released metal ions and metal hypersensitivity-induced allergic dermatitis, pain, and implant failure [43].

### 2.3. Aseptic Knee Implant Loosening

Implant wear debris is known to be an initiating event for aseptic implant loosening [44]. A significant number of failed knee implants display perivascular lymphocytic infiltration indicative of an adaptive immune response; however, the question remains if this finding truly suggests a causal relationship between metal hypersensitivity and implant failure [45]. Several studies have indicated potential pathomechanisms for aseptic implant loosening secondary to hypersensitivity. The common mechanism is the secretion of proinflammatory cytokines induced by metal ions; these lead to the formation of osteolytic lesions in the bone surrounding metallic implants [30]. The NALP3 inflammasome within macrophages has been shown to be a critical instigator and mediator of orthopaedic implant-induced osteolysis [46]. The osteolytic mechanism that responds to implant debris likely involves the receptor activator of NF-*κβ* (RANK) and the receptor activator of NF-*κβ* ligand (RANKL) pathway, as well as osteoprotegerin and IL-18 [47]. Titanium has been shown to directly increase the expression of RANKL, macrophage colony stimulating factor, and TNF-*α* [27]. Titanium ion-induced expression and secretion of CCL17 and CCL22, as well as upregulation of the CCR4 receptor, result in osteoclast precursor recruitment to the periprosthetic region, whereas the previously induced cytokines promote osteoclast differentiation and activation [27]. Chronic periprosthetic inflammation and the induction of macrophage-mediated aseptic osteolysis may ultimately result in implant loosening and failure [6].

## 3. Patients

### 3.1. Published Case Studies

The majority of information regarding hypersensitivity reactions following TKA is derived from a limited number of case studies. The authors reviewed all available published reports of metallic knee implant-associated hypersensitivity reactions; details of the individual case studies are outlined in Table 3 and summarized in the following paragraph [12, 48–55].

In the 28 reported cases of metal hypersensitivity reactions after TKA, 23 of the patients were female [12, 48–55]. Seven patients had a history of metal hypersensitivity before the arthroplasty [52–54]. The orthopaedic implants associated with reactions were composed of various combinations of metals, including cobalt, chromium, molybdenum, copper, nickel, titanium, aluminum, and vanadium [12, 48–55]. Most patients presented with varying degrees of periprosthetic irritation, although one patient presented with systemic dermatitis. There was a report of decreased range of motion in three patients; in one of these patients, arthroscopy demonstrated a lymphoplasmacellular fibrinous tissue consistent with a delayed-type hypersensitivity reaction [48, 50, 55]. Skin patch testing was performed in all but 1 of the 28 patients, with 18 of the patients having positive results [12, 48, 49, 51–55]. Lymphocyte transformation testing (LTT) was performed in six patients, including the one patient who was not tested with a skin patch [50, 54]. Five patients had positive lymphocyte transformation tests. Aseptic implant failure was observed in two patients [53, 54]. Both patients experienced implant failure with the initial replacement and with the first revision procedure. After the second revision procedure using hypoallergenic implants, the dermatologic symptoms resolved and the implants remained stable. It is important to note that there have been two published case studies in which both patients had a history of metal hypersensitivity but did not develop any adverse hypersensitivity reactions following TKA even though the implants contained components to which the patients were allergic [13, 57].

### 3.2. Risk Factors

The risk of becoming sensitized to metal varies largely depending on an individual's exposure. Several risk factors for developing metal hypersensitivity have been identified, including age, gender, and occupation. With increasing age, there is a decreased risk of developing nickel hypersensitivity [36, 37, 39]. This may be attributed to decreased lifetime exposure of older individuals to metallic costume jewelry [36]. Exposure to costume jewelry (particularly earrings) may also account for the sex discrepancy that is observed in patients with metal hypersensitivity [36, 58]. In general, women are at an increased risk for developing hypersensitivity to several metals [8]. Epidemiologic studies place the rate of nickel sensitization in women between 17% and 32%, whereas the sensitization rate in men is significantly lower—between 3 and 10% [36, 37, 58]. The rate of cobalt sensitization is 11.2% in women and 8.4% in men [58]. The literature identifies only one metal to which men are more sensitized than women. The rate of chromium sensitization in men is 10.1% whereas in women it is 7.9% [58]. Interestingly, these differences correspond to metal exposures that occur in traditionally sex-specific occupations. Chromium hypersensitivity is associated with concrete exposure in the construction industry, leatherwork and tanning, and occupations involved in cleaning [32, 36, 58]. Sensitization to cobalt is also associated with occupations involved in cleaning and leatherwork, as well as hairdressing and professions in the textile industry [58]. Nickel sensitization is associated with occupations in healthcare, agriculture, mechanics, and metalwork [58].

A history of metal allergy in patients may also be a significant risk factor that should be taken into consideration before TKA. Granchi et al. reported the implant failure rate that was four times greater in patients with a self-reported history of preoperative metal allergy compared with patients who did not have a metal allergy [8].

### 3.3. Presentation

As with all pathological processes, hypersensitivity reactions to metallic knee implants can present several ways. Metal hypersensitivity may result in localized or systemic allergic dermatitis, loss of joint function, implant failure, and patient dissatisfaction [32]. Hypersensitivity reactions after TKA are most commonly present in the first few postoperative months as pruritic, erythematous, eczematous, edematous, sometimes painful, and sometimes exudative lesions in the periprosthetic region [12, 48–55]. In patients with a TKA implant containing metal, the clinician should consider metal hypersensitivity when dermatologic allergic symptoms are reported. Furthermore, metal hypersensitivity should be considered in such patients when they present with arthralgia, when periprosthetic radiolucent lines appear, or when aseptic implant loosening is observed [59].

## 4. Screening

### 4.1. Patient History

The patient history plays an invaluable role in making a diagnosis in every field of medicine. When diagnosing metal hypersensitivity, patient-reported history of a metal allergy should not be ignored. In a study of 22 patients with a self-reported history of metal hypersensitivity, skin patch test results were positive in 19 patients [13]. A similar study found positive gold standard skin patch test results in 68% of the patients with a history of metal allergy [9]. It should be considered, however, that the patient history and gold standard skin patch test results do not necessarily correlate, particularly in patients with a history that is negative for metal hypersensitivity [60]. Frigerio et al. reported that patient history taking is appreciably less reliable than gold standard testing in determining metal sensitization; the sensitivity of patient history is 85.5% and the specificity is 83.5% [6].

### 4.2. Patch Testing

Cutaneous patch testing is the gold standard for in vivo evaluation of delayed hypersensitivity reactions [5]. As previously discussed, metal hypersensitivity to orthopaedic implants displays distinct characteristics of delayed-type hypersensitivity. Many physicians believe that the patch test method is an acceptable approach for evaluating hypersensitivity to orthopaedic joint implant components [8].

In the general population, there has been a significant increase in the number of positive patch test results over the past four decades; this increase is most likely attributable to a substantial rise in the number of metals tested [61]. Despite this increase in the general population, patients who have undergone TKA remain significantly more likely to have a positive patch test [8, 61]. The rate of positive patch test results to metals is even greater in patients with metal-on-metal implants and in those with a failed prosthesis [61]. This correlates with the finding that positive patch tests are associated with shorter implant lifespans [19].

There are several advantages to evaluating metal hypersensitivity with patch tests following total knee arthroplasty. In a published report of 21 patients with positive patch tests to metals, none experienced hypersensitivity reactions after TKA with hypoallergenic implants [9]. These findings can be viewed as support for the argument that preoperative patch testing potentially prevents significant morbidity [5]. Practical advantages of cutaneous patch testing include ease of performance, rapidity of results, the scope of evaluation, and widespread availability [19, 43]. As with all ideal testing methods, the risk to the patient in patch testing is generally quite low [5].

Without disputing the numerous advantages of patch testing, questions remain regarding the propriety of patch testing in evaluating implant-induced hypersensitivity reactions. Some investigators cite the differences in antigen-presenting cells in superficial and deep tissues as a cause for doubt; this doubt leads to questions regarding the validity of cutaneous test results as they relate to periprosthetic tissue [7, 8, 14, 25]. Other investigators have noted that, despite a strong correlation, no causal relationship has been definitively established between dermal reactions and implant failure [50]. Analyses have determined that patch testing results, although valuable in patients with suspected hypersensitivity, had no predictive value for complications when performed prior to arthroplasty [8, 61]. These arguments have contributed to the reluctance of orthopaedic surgeons to use cutaneous patch testing in routine orthopaedic practice [3, 25, 62].

Although primarily theoretical, a potential disadvantage of patch testing is that the process of in vivo patch testing could potentially induce sensitization in a previously nonsensitized patient [25]. If this occurred in a patient who had previously undergone arthroplasty, it could place the patient at risk of significant morbidity secondary to an iatrogenically induced hypersensitivity.

Patch testing remains the gold standard for evaluation of delayed-type hypersensitivity. Its preoperative use should strongly be considered in patients with a history of metal allergies and its postoperative use in patients presenting with either suspected metal hypersensitivity or implant failure in the absence of infection [5, 61].

### 4.3. Lymphocyte Transformation Testing

Lymphocytes transformation testing (LTT) can be used as an alternative method to determine metal sensitivity in a patient. This in vitro test measures the proliferation of lymphocytes from a patient's peripheral blood in the presence and absence of a potential allergen [5]. It has been suggested for use when patch testing provides questionable results [5, 19].

LTT has several important advantages compared to cutaneous patch testing. In determining a patient's reactivity to metal, LTT offers greater sensitivity than patch testing [40]. Because of the nature of the LTT construct, highly quantifiable and reproducible measures of sensitivity are available; no such objective results exist for patch testing [43, 63]. Unlike patch testing, LTT cannot induce sensitization because it is performed in vitro [63]. The greatest risk to a patient with LTT is venipuncture. Most encouragingly, a prospective study using LTT prior to arthroplasty indicated that it may be effective as a preoperative screening tool for metal hypersensitivity [24].

LTT remains largely impractical for routine clinical use. The availability of laboratories equipped to perform this test is limited; such facilities are primarily restricted to university settings [5, 43]. Because few allergens are tested, LTT is much more restricted in breadth of evaluation compared to patch testing [5]. Although LTT is known to have a greater sensitivity compared to patch testing, the precise sensitivity and specificity of LTT have not yet been established [25, 40].

### 4.4. Other Screening Options

Beecker et al. reported on a case study of a patient with a known history of metal hypersensitivity and established positive patch test reactions to nickel and cobalt [52]. The patient underwent subcutaneous embedding of cobalt and titanium implants [52]. At 6 weeks, no reaction to the implanted metals was noted; however, after the patient underwent TKA of the left knee, periprosthetic allergic contact dermatitis developed [52]. One year later, the patient underwent total knee arthroplasty of the right knee [52]. Periprosthetic hypersensitivity reactions again developed [52]. No established guidelines exist regarding the depth or the duration of subcutaneous metal implantation as a screening test for hypersensitivity. This patient's outcome suggests the poor sensitivity of this method for at least the first 6 weeks of subcutaneous metal implantation. This approach is not recommended.

### 4.5. Timing

Standard screening of all patients for metal hypersensitivity prior to total knee arthroplasty is not appropriate [43]. In addition to generating unnecessary expense, a large portion of the general population tests positive for nickel allergy despite the absence of hypersensitivity symptoms [36–39]. Therefore, preoperative testing is only indicated in patients with a history of either metal allergy or previous aseptic orthopaedic implant failure [9, 43]. Some additional guidelines for preoperative patch testing exist. Schalock et al. recommend using a baseline series based on the patient's place of residence [5]. Various national and international dermatologic organizations have established appropriate baselines. A history of hypersensitivity to metals that are not included in the baseline series warrants expansion of the testing parameters [5].

Postoperative testing for metal sensitization is appropriate in a select group of patients. Such testing should be considered if a patient presents with recent onset periprosthetic allergic contact dermatitis or arthralgia and when radiolucent lines appear on radiographs or implant loosening is observed [5, 59]. Infectious etiologies of these symptoms should be ruled out first.

## 5. Prevention and Management

### 5.1. Case Study Follow-Up

In the case studies previously discussed, patients with metal hypersensitivity were managed with a variety of approaches. The details of individual case studies are outlined in Table 3. The dermatologic symptoms of 15 patients were resolved completely with the use of topical corticosteroid [12]. The bilateral intermittent cutaneous reactions of one patient were managed with topical treatment over an 8-year course with topical treatment [52]. Ten patients were treated with surgical revision utilizing hypoallergenic prostheses; the revision implants included four titanium-based implants, two zirconium-nickel coated implants, one zirconium-ceramic alloy implant, one titanium and ceramic implant, one cobalt and ceramic alloy coated implant, and one ceramic implant [48, 50, 51, 53–55]. One case study did not mention the treatment approach or patient outcome [49].

### 5.2. Approach Considerations

**Figure 1:**
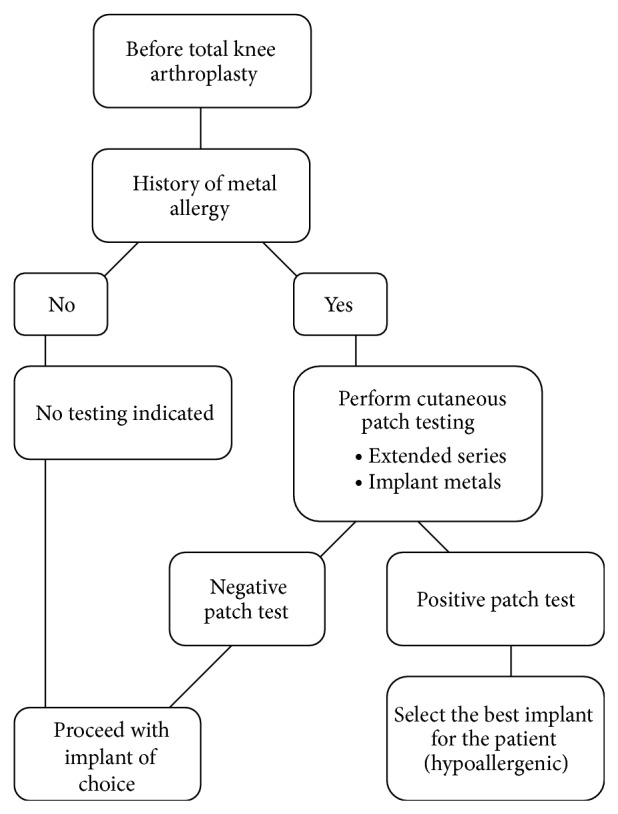


**Figure 2:**
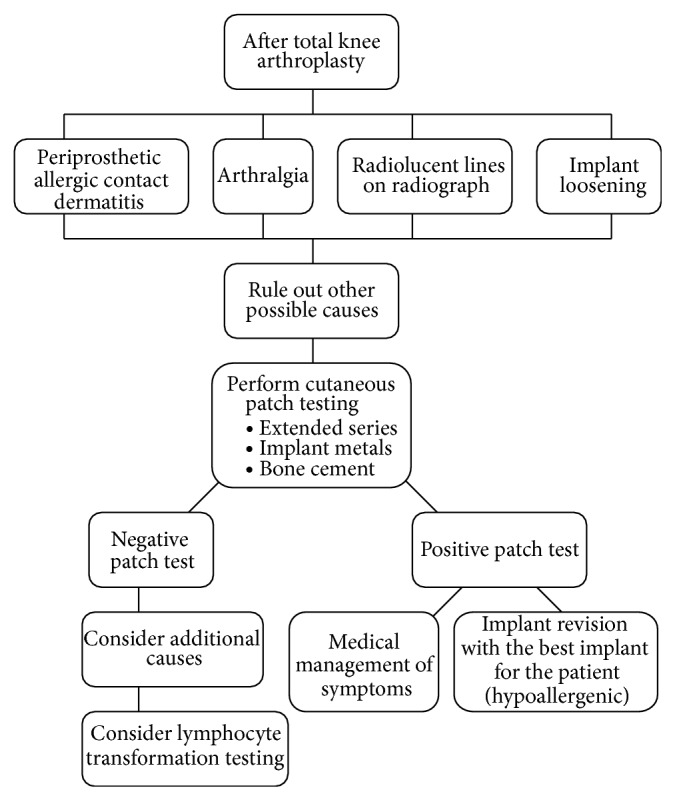


Several diagnostic algorithms have been suggested for orthopaedic patients with suspected metal hypersensitivity [5, 61]. Comprehensive diagnosis and treatment algorithm are presented in Figures 1 and 2, respectively. Postoperative intervention should follow positive patch test results only when patients are symptomatic or the implant demonstrates clear evidence of failure [5, 9]. Consideration also should be given to nonmetal allergic reactions after TKA. Benzoyl peroxide found in bone cement may cause delayed hypersensitivity reactions [5, 64, 65].

There are many options when considering implants for patients who are sensitive to metal. The ideal prosthesis does not contain metals to which the patient has been sensitized [5]. Because titanium sensitivity is rare, it has been suggested that titanium implants be used in all TKA patients. These implants, however, are inappropriate for most patients because they are typically unnecessary and are substantially more expensive [43]. Patients who are sensitive to metal, even those with titanium-coated prostheses, experience greater functional limitations and decreased quality of life compared with their nonallergic counterparts with standard implants [56]. The most important consideration is whether aseptic loosening has occurred secondary to metal hypersensitivity [66].

## 6. Conclusion

Currently available evidence demonstrates both incidence and probable mechanisms for metal hypersensitivity reactions after total knee arthroplasty. This is an uncommon complication but must be recognized to ensure the health and satisfaction of patients. Some studies acknowledge the correlation but do not identify a causative relationship. However, based on the current evidence, the authors of this paper believe in a likely casual association between metallic knee implants and hypersensitivity reactions that can potentially lead to aseptic implant failure.

## Figures and Tables

**Table 1:** Hypersensitivity reactions after total joint arthroplasty reported in prospective studies.

Prospective study title	Publication	Author	Results
Sensitivity to implant materials in patients undergoing total hip-replacement	J Biomed Mater Res	Granchi et al. [19]	Patch test unable to differentiate stable versus unstable implants, equivalent implant lifespan in metal patch +; 10 yr survival for metal patch + 44% versus patch − 47%; poor survival for cement patch +
Allergy to components of total hip arthroplasty before and after surgery	Ital J Orthop Traumatol	Cancilleri et al. [20]	10/66 THA patch + (1/12 w/aseptic loosening patch +), 2/41 preop. patch +; hypersensitivity may play role in loosening, but likely small
Metal sensitivity in patients with metal-to-plastic total hip arthroplasties	Acta Orthop Scand	Carlsson et al. [11]	9/112 patch + preop., 12/112 patch + postop.; all complications except 1/246 explained by reasons other than hypersensitivity
Allergy in hip arthroplasty	Contact Dermatitis	Waterman and Schrik [21]	13/85 patch + preop. (13 metal), 25/85 patch + postop. (23 metal, 2 cement), 0/10 loose THA patch +; no evidence to suggest loosening because of hypersensitivity
The development of metal hypersensitivity in patients with metal-to-plastic hip arthroplasties	Contact Dermatitis	Nater et al. [22]	0/66 patch + preop., 4/66 patch + MOP conversion postop.; no clinical sequelae, no need to test
Metal sensitivity in patients with orthopedic implants: a prospective study	Contact Dermatitis	Frigerio et al. [6]	16/72 (22%) preop. + patch or LTT, (19/72 (29%) postop. (5 conversions of 72 total)); if preop. history insufficient, recommend for screening tests
Metal sensitivity before and after total hip arthroplasty	J Bone Joint Surg Am	Deutman et al. [23]	10/173 patch + preop., 4/66 converted patch + postop. MOP; no conclusion
Metal sensitivity in patients undergoing hip-replacement	J Bone Joint Surg Br	Rooker and Wilkinson [10]	6/69 patch + preop. MOP, only 1/54 patch + postop.; patch + may be effect not cause, no need to screen in MOP
The effect of patch testing on surgical practices and outcomes in orthopedic patients with metal implants	Arch Dermatol	Mesinkovska et al. [9]	31 with history of hypersensitivity preop., 21 patch +, all did well with “allergen-free” implants; 41 suspected of hypersensitivity w/TJA, 10 patch +, 6/10 had resolution of symptoms with allergen free implant; recommend patch testing in those with history
Screening for symptomatic metal sensitivity: a prospective study of 92 patients undergoing total knee arthroplasty	Biomaterials	Niki et al. [24]	24/92 TKA were mLST+ preop., 5/24 developed eczema, Cr + in eczema patients but not in others; screening indicated

Prospective study summary			Preop. patch/LTT +: 56/618 (9.1%), postop.: 73/521 (14.1%) Conversion of patch/LTT preop. to postop.: 56/618 (9.1%) preop. versus 73/521 (14.0%) postop.

**Table 2:** Hypersensitivity reactions after total joint arthroplasty in retrospective studies.

Retrospective study title	Publication	Author	Results
Contact allergy to metals and bone cement components in patients with intolerance of arthroplasty	Dtsch Med Wochenschr	Eben et al. [15]	In cemented TJA: 22/66 symptomatic pts. patch +, asymptomatic patch + 3/26
Allergy to metals as a cause of orthopaedic implant failure	Int J Occup Med Environ Health	Kręcisz et al. [16]	14 poor implants, 8 patch + (7 ni, 6 cr), 3 underwent revision and improved
Early osteolysis following second-generation metal-on-metal hip-replacement	J Bone Joint Surg Am	Park et al. [17]	8/9 MoM w/osteolysis patch + to Co, 2/9 w/o osteolysis patch +; retrospective
Sensitivity to metal as a possible cause of sterile loosening after cobalt-chromium total hip-replacement arthroplasty	J Bone Joint Surg Am	Brown et al. [18]	0/20 loose MoM patch + (1977)
Metal sensitivity as a cause of bone necrosis and loosening of the hip prosthesis in total joint replacement	J Bone Joint Surg Br	Evans et al. [2]	9/14 w/loose joints patch +, 0/24 w/stable joints
Incidence of metal sensitivity in patients with total joint replacements	Br Med J	Elves et al. [1]	15/23 failed TJA patch +, 4/27 stable patch +, 8/13 w/derm rxn were patch +
Dermatitis on the knee following knee replacement: a minority of cases contact allergy to chromate, cobalt, or nickel but a causal association is unproven	Contact Dermatitis	Verma et al. [12]	7 of 15 patients w/cutaneous symptoms patch +
Metal sensitivity in patients with metal-to-plastic total hip arthroplasties	Acta Orthop Scand	Carlsson et al. [11]	13/134 MOP patch + postop.; unsure if hypersensitivity caused by THA, but, in pts. w/Hx of allergy, proceed w/caution
Retrospective evaluation of patch testing before or after metal device implantation	Arch Dermatol	Reed et al. [13]	5/22 with history of hypersensitivity preop. patch +, 0/22 referred for patch test postop. were patch +
Lymphocyte responses in patients with total hip arthroplasty	J Orthop Res	Hallab et al. [14]	More + LTT and cytokine release in THA, and esp. in loose THA

Retrospective study summary			Revised: 33/138 (23.9%) patch +, 44/303 (14.5%) patch + in stable TJA Failed/loose: 113/261 (43.3%) patch +, 32/146 (21.9%) patch + in TJA Total: 146/399 (36.6%) patch +, 76/449 (16.9%) patch − 10/16 (62.5%) revised TJAs LTT +

**Table 3:** Summary of case reports of metallic knee implant associated hypersensitivity reactions.

	Number of implants	Patient gender	History of metal allergy	Implant components	Presenting signs and symptoms	Patch test result	LTT result	Initial implant outcome	Treatment	Revision implant components
Beecker et al. [52]	2 (1 patient)	Female	Yes	Cobalt-chromium alloy	Erythema, edema, heat, eczema	Cobalt, nickel		Stable	Triamcinolone, diphenhydramine	
Bergschmidt et al. [48]	1	Female		Cobalt-chromium alloy	Arthralgia, heat, decreased ROM	Nickel sulfate, palladium chloride		Stable	Revision	Ceramic, titanium
Dietrich et al. [54]	4	Female	Yes	Cobalt-chromium-molybdenum alloy	Arthralgia, erythema, edema	Cobalt, nickel	Cobalt, nickel	Stable (3) Failure (1)	Revision	Titanium
Gao et al. [55]	1	Male	No	Cobalt-chromium-molybdenum alloy	Eczema, systemic dermatitis, decreased ROM	Chromium		Stable	Revision	Zirconium-niobium alloy
Handa et al. [49]	1	Male	No	Copper-chromium alloy	Eczema, exudate	Cobalt, copper		Stable		
Oiso et al. [51]	1	Male		Cobalt-chromium alloy	Erythema, edema, arthralgia, fever	Cobalt, chromium, nickel, manganese		Stable	Revision	Ceramic, titanium
Thomsen et al. [50]	1	Female			Arthralgia, eczema decreased ROM		Negative	Stable	Revision	Zirconium-nitride coating
van Opstal and Verheyden [53]	1	Female	Yes	Titanium-aluminum-vanadium	Arthralgia, eczema, edema	Negative		Failure	Revision	Zirconium alloy
Verma et al. [12]	15	Female (13) Male (2)	No	Cobalt-chromium alloy	Eczema	Nickel (4) Chromium (2) Cobalt (1)		Stable	Topical corticosteroid	
